# Metabolomic Profiles and Biopharmaceutical Properties of *Petrosimonia brachiata* and *P. nigdeensis* from Turkey

**DOI:** 10.3390/plants13152073

**Published:** 2024-07-26

**Authors:** Marco A. De Gregorio, Leilei Zhang, Mohamad Fawzi Mahomoodally, Gokhan Zengin, Sharmeen Jugreet, Evren Yildiztugay, Andrea Fiorini, Luigi Lucini

**Affiliations:** 1 Department of Sustainable Food Process, Università Cattolica del Sacro Cuore, 29122 Piacenza, Italy; marcoarmando.degregorio@unicatt.it (M.A.D.G.); luigi.lucini@unicatt.it (L.L.); 2 Institute of Research and Development, Duy Tan University, Da Nang 550000, Vietnam; mohamadfawzimahomoodally@duytan.edu.vn; 3 School of Engineering & Technology, Duy Tan University, Da Nang 550000, Vietnam; 4 Department of Biology, Science Faculty, Selcuk University, Konya 42130, Turkey; 5 Department of Health Sciences, Faculty of Medicine and Health Sciences, University of Mauritius, Réduit 80837, Mauritius; sharmeenjugs@gmail.com; 6 Department of Biotechnology, Science Faculty, Selcuk University, Konya 42130, Turkey; eytugay@gmail.com; 7 Department of Sustainable Crop Production, Università Cattolica del Sacro Cuore, 84, 29122 Piacenza, Italy; andrea.fiorini@unicatt.it

**Keywords:** *Petrosimonia brachiata*, *Petrosimonia nigdeensis*, flavonoids, phenolics, antioxidant, enzyme inhibition

## Abstract

Halophytic plants possess a huge range of active constituents and medicinal benefits. In this study, extracts (water, ethanol, ethyl acetate, dichloromethane, and n-hexane) of two halophytes of the genus *Petrosimonia* (*P. brachiata* and *P. nigdeensis*) were investigated for their phytochemical profiles and pharmacological properties. The phytochemical profiles of both species were investigated using an untargeted metabolomics approach based on high-resolution mass spectrometry. The two species show different polyphenolic profiles and these are influenced by the different extraction solvents used. The same extracts were used for different bioactivity assays. The results show that all extracts yielded total flavonoid and phenolic contents of 11.14–24.22 mg GAE/g and 3.15–22.03 mg RE/g, respectively. While extracts of both species demonstrated a radical scavenging ability in the ABTS assay (16.12–98.02 mg TE/g), only the polar and moderately polar extracts (water, ethanol, and ethyl acetate) showed scavenging potential in the DPPH assay (4.74–16.55 mg TE/g). A reducing potential was also displayed by all extracts in the CUPRAC and FRAP assays (26.02–80.35 mg TE/g and 31.70–67.69 mg TE/g, respectively). The total antioxidant capacity of the extracts ranged from 0.24 to 2.17 mmol TE/g, and the metal chelating activity ranged from 14.74 to 33.80 mg EDTAE/g. The water extracts possessed a higher metal chelating power than the other extracts. All extracts acted as inhibitors of acetylcholinesterase (0.16–3.85 mg GALAE/g) and amylase (0.11–1.28 mmol ACAE/g). Moreover, apart from the water extracts, the other extracts also showed anti-butyrylcholinesterase activity (0.73–2.86 mg GALAE/g), as well as anti-tyrosinase (36.74–61.40 mg KAE/g) and anti-glucosidase (2.37–2.73 mmol ACAE/g) potential. In general, the water extracts were found to be weak inhibitors of the tested enzymes, while the ethanol extracts mostly showed an inhibitory effect. The obtained findings revealed the antioxidant and enzyme inhibitory properties of these two species and demonstrated that the solvent type used affected the pharmacological properties of the extracts and hence, can be useful to further investigate the active constituents yielded in the extracts and understand the mechanisms involved.

## 1. Introduction

Halophytes display distinct morphological and physiological adaptations to thrive in saline ecosystems [1]. Their multifactorial adaptive responses comprise a complex network of biochemical mechanisms and abundant bioactive molecules, such as phenolic compounds, glycosides, polysaccharides, lipids, alkaloids, and other related compounds [2]. Indeed, a broad range of secondary compounds of economic interest is present in halophytes, and many of these are limited to halophytic species [3].

The ethnobotanical literature provides evidence that due to their phytochemical richness, halophytic medicinal plants have extensively been used for treating numerous infectious diseases, particularly in developing countries where traditional medicine is still employed as an initial approach to treat minor illnesses [4,5]. A variety of species have traditionally been used as medicines against seven different types of disease conditions like respiratory, skin, digestive, fever, pain, toothache, genito-urinary, and others [6]. Several species of salt-tolerant plants have also been documented to possess a wide range of ethnomedicinal uses for human parasitic diseases. In fact, the antiprotozoal and anthelmintic properties of halophytes have previously been determined using in vitro and in vivo methods, and bioactive metabolites that may be associated with such properties have been established [7]. Thus, interest in halophytic plants has grown during recent years, given their economic potential, and has been supported by studies revealing them to be potential candidates for medicinal purposes [8].

The genus *Petrosimonia*, a member of the Chenopodiaceae family, includes between 11 and 15 halophytic species that are present in Southeast Europe, as well as in Central and Southwest Asia [1]. The plants (*P. branchiata* and *P. nigdeensis*) are erect and generally more than 10 cm and branched. Their leaves are almost 2–5 cm in size. The plant differentiates itself with some characteristics. *P. branchiata* has opposite leaves and five perianth segments. However, *P. nigdeensis* has alternate upper leaves and three perianth segments [9]. They are well known to possess medicinally important constituents [10,11,12,13]. While several of their species have been investigated for their salt tolerance mechanisms [14,15,16], data on their pharmacological importance are quite limited. Considering the Halophytes genus, Shehab et al. [17] evaluated the richness in secondary metabolites in four different species. In particular, the phenolic profiles were evaluated. The results suggest that the phenolic profiles are species-specific even though all plants live in the same environmental conditions. Nurpeisova et al. [10] conducted the first study on the phenolic profile of *P. sibirica*. They found this species to be a rich source of saponins and flavonoids. The extraction was performed exclusively using ethyl alcohol. Extracts of other species of *Petrosimonia* have also been analyzed for their phytochemical contents. For instance, a phytochemical screening of *P. sibirica* showed the presence of several primary and secondary metabolites. The composition of 20 amino acids and 8 fatty acids of *P. sibirica* were established. The major amino acids were alanine, glutamic acid, aspartic acid, arginine, tyrosine, and proline, while the main fatty acids were oleic and linoleic acids. In addition, 70% ethyl alcohol was found to be the most suitable solvent, and other factors such as the solid–solvent ratio (1:6–8), extraction time (3 days), and temperature (20–25 °C) were determined. Additionally, the *P. sibirica* plant was found to be a rich source of saponins and flavonoids such as quercetin 3-O-β-D-glucopyranoside (isoquercitrin) [10]. Furthermore, a phytochemical analysis of the aerial part of *P. glaucescens* revealed the presence of polysaccharides, flavonoids, organic acids, saponins, alkaloids, and coumarins, including condensed tanning agents [12]. There is no additional information available in the literature regarding the extraction efficiency of different solvents for *Petrosimonia* species. Regarding the ethnobotanical uses of the members of the *Petrosimonia* genus, *P. branchiata* is widely used to treat fever, headache, and stomach aches [18].

Hence, this study was aimed at investigating the metabolomic profiles and biopharmaceutical properties of different extracts (water, ethanol, ethyl acetate, dichloromethane, and n-hexane) of two *Petrosimonia* species (*P. brachiata* and *P. nigdeensis*) from Turkey.

## 2. Results and Discussion

### 2.1. Total Phenolic and Flavonoid Contents

The determination of the total phenolic and flavonoid contents (TPC and TFC) of medicinal plants in preliminary studies is considered to be important in testing the bioactive contents of prepared extracts. In fact, numerous studies have used the Folin–Ciocalteu and aluminum chloride colorimetric assays for determining the TPC and TFC of various plant extracts [19,20,21]. Furthermore, the existence of numerous phenolic families in plants having different chemical structures and polarities results in the use of a wide range of extraction solvents (water, acetone, ethanol, methanol, or their mixtures with water). Nevertheless, despite the interests of several researchers in the extraction of polyphenols, there is no single solvent that can be regarded as a standard since it is usually different for different plant matrices [22]. For instance, polar solvents are usually utilized to extract phenolic compounds, glycosides, and saponins, while non-polar solvents are used for fatty acid and steroid extractions. Many studies have even reported the influence of different solvents on the content of secondary metabolites, as well as on their antioxidant capacity [23,24]. Herein, different degrees of solvent polarity were used, considering water, ethanol, ethyl acetate, dichloromethane, and n-hexane.

In the present study, the general TPC spectrometric determination yielded in the range of 11.14 to 23.91 mg GAE/g and 14.09 to 24.22 mg GAE/g, whereas TFC was yielded in the range of 14.09–24.22 mg RE/g and 3.15–22.03 mg RE/g in *P. brachiata* and *P. nigdeensis*, respectively. While the *P. nigdeensis* ethanol extract showed both the highest TPC and TFC compared to the other *P. nigdeensis* extracts, the *P. brachiata* ethyl acetate and ethanol extracts showed the highest TPC and TFC, respectively, compared to other *P. brachiata* extracts (Table 1). The total flavonoid (2.41 mg CE g^−1^ DW) and polyphenol (4.06 mg GAE g^−1^ DW) contents were also detected in *P. triandra* [8]. The TPC and TFC of the methanol, ethanol, water, n-hexane, and dichloromethane extracts of *P. nigdeensis* stem and fruit/leaf were also tested in a previous study by Asan-Ozusaglam et al. [25]. The authors used a Soxhlet apparatus for extraction with various solvents. In their study, the stem dichloromethane extract yielded the highest TPC and TFC (40.89 µg GAE/mg extract and 57.55 µg QE/mg extract, respectively), unlike in the present study. In addition to the differences in extraction technique, the differences can be explained by the geographical and climatic conditions in the plant collection areas.

Based on the obtained results, the total phenolic and flavonoid contents depended on the polarity of the extraction solvents used. In general, ethanol and ethyl acetate contained more phenolics compared to the other solvents. Ethyl acetate is a medium-polarity solvent, making it effective at dissolving a wide range of polar and non-polar compounds. In addition, it has the ability to penetrate plant tissues and solubilize phenolic compounds effectively. Ethanol is a polar solvent and is generally recognized as safe (GRAS) by regulatory agencies and is commonly used in the food and pharmaceutical industries [26]. Based on this fact, we can hypothesize that ethanol can be used as a solvent for manufacturing pharmaceutical applications with *Petrosimonia* species. However, in recent years, the spectrophotometric tests for total phenolic and flavonoid content have some drawbacks. In particular, not only a specific group of plant substances, but also other substances (particularly peptides) can react with the reagents used, which can lead to incorrect results [27]. Therefore, the obtained results have to be confirmed using a chromatographic technique.

### 2.2. Metabolomic Profiling of Two Petrosimonia Species (P. brachiata and P. nigdeensis)

The effect of different extraction solvents on the yield of phenolic compounds in the two species of *Petrosimonia* was studied using an untargeted metabolomic approach. The phenolic profile allowed us to record 100 features, characterized mainly by low-molecular-weight (LMW) phenolic compounds—the most frequent class of phenolic compound—with 33 features (7 of these are tyrosol derivates), followed by flavones (16 metabolites), phenolic acids (19 metabolites), anthocyanins (14 metabolites), flavonols (9 metabolites), lignans (5 metabolites), and stilbenes (4 metabolites). The whole list of polyphenols annotated is provided in the Supplementary Materials (Supplementary Table S1), which provides comprehensive information on their retention time and mass spectrum. To assess the effect of the different extraction solvents, a semi-quantitative analysis of the main phenolic classes, expressed as µg phenolic equivalents g^−1^ dry matter (DM), was carried out and is reported in Table 2. This semi-quantitation was performed for both *Petrosimonia* species. Under our experimental conditions, the most abundant classes of polyphenols were phenolic acids (*P. brachiata* 440.43 µg/g and *P. nigdeensis* 101.93 µg/g) and LMW compounds (*P. brachiata* 206.69 µg/g and *P. nigdeensis* 334.93 µg/g). Specifically, 68% of the phenolic acids contained in *P. brachiata* were extracted in water, while 30.5% of LMW compounds in ethanol. In the case of *P. nigdeensis*, 25% of phenolic acids were extracted in the same amount in water, ethanol, and ethyl acetate.

### 2.3. Multivariate Discrimination Analysis of Two Petrosimonia Species

**Figure 1:**
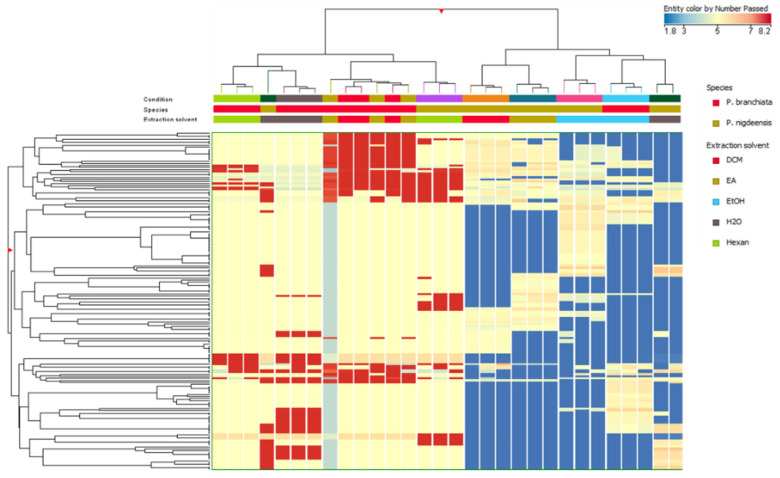
Unsupervised hierarchical clustering analysis (HCA) performed using Log2 fold change median normalized values of each annotated compound based on the tested species/solvents.

**Figure 2:**
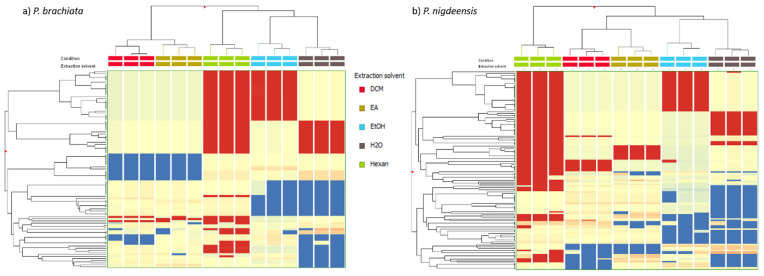
Unsupervised hierarchical clustering analysis (HCA) performed using Log2 fold change median normalized values of each annotated compound. In the legends are reported the extraction solvents—dichloromethane (DCM), ethyl acetate (EA), ethanol (EtOH), water, and n-hexane.

The discrimination analysis of the two different *Petrosimonia* species was carried out using two approaches. The first approach consisted of an unsupervised hierarchical cluster analysis (HCA) that clusters samples based on their similarities and/or dissimilarities considering phenolic compounds extracted using different solvents (Figure 1). As reported in the figure, HCA reported four different clusters, highlighting the majority effect of species factor on the phenolic profile compared to solvent extraction. To better investigate this aspect, two different HCA related to species were elaborated. (Figure 2). For both species, three different branches were identified, firstly represented by water- and ethanol-extracted samples, then by ethyl acetate (EA) and dichloromethane (DCM), and finally by samples extracted with n-hexane.

**Figure 3:**
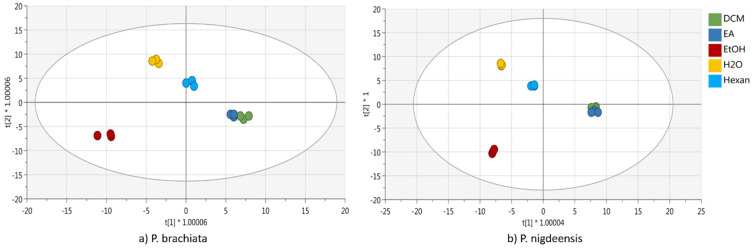
Supervised orthogonal projections to latent structures discriminant analysis (OPLS-DA) score plot of *P. brachiate* (**a**) and *P. nidrigeensis* (**b**) built according to the phenolics compounds in different extraction solvents. In the legends are reported the extraction solvents—dichloromethane (DCM), ethyl acetate (EA), ethanol (EtOH), water, and n-hexane.

The second approach adopted was the supervised Orthogonal Projection to Latent Structure Discriminant Analysis (OPLS-DA; Figure 3). In the figure, the OPLS-DA models for *P. brachiata* (Figure 3a) and *P. nigdeensis* (Figure 3b) are reported. The OPLS-DA model generated for the *P. brachiata* samples confirmed the data produced using HCA, reporting a high discrimination performance between different kinds of extraction solvents. The score plot generated was characterized by high performance parameters, such as goodness of fit (R2) and the prediction capacity of this model (Q2) at 0.990 and 0.941, respectively. Moreover, the model was validated through cross-validation (CV-ANOVA *p*-value < 0.05) and permutation tests in order to exclude model overfitting. To identify the most discriminant compounds contributing to the differences outlined in the OPLS-DA model, the variable importance in projection (VIP) compounds were selected with a VIP score ≥ 1.2 (Table 3), inclusive of compound classification, VIP score ± standard errors, and log fold change obtained via a pairwise comparison between different extraction solvents and water.

The OPLS-DA model generated for *P. nigdeensis* was able to discriminate all five extraction solvents perfectly with two latent vectors. Indeed, the score plot generated was characterized by high performance parameters (R^2^ = 0.995 and Q^2^ = 0.93), as well as without model overfitting. The list of VIP biomarkers (VIP ≥ 1.2) extrapolated from the model is reported in Table 4, inclusive of compound classification, VIP score ± standard errors, and log fold change obtained via a pairwise comparison between solvents and water (control).

For both species, chemometrics analyses confirmed the importance of the extraction solvent in relation to the different phenolic profiles obtainable [28]. This may depend on the heterogeneity of the classes we call polyphenols and the modifications they may undergo (methylation, sulfonation, and glycosylation) [29]. So, in general, the relative hydrophilicity or lipophilicity of polyphenols depends on the number of contained hydroxyl groups [30].

Considering the two species under analysis, the VIP compound classes revealed by the OPLS are markedly different. *P. brachiata* appears to be the species with the highest complexity of chemical species. Flavones, alkylphenols, and alkylmethoxyphenols, primarily surfactants and scaffolds of pharmaceutical preparations, are notable [31,32]. Flavonoids, in particular, are considered important scaffolds, with their structure often referred to as the “skeleton key” [33]. This designation arises because flavones are an essential core for many compounds that act on various targets, eliciting different pharmacological properties through various substitution patterns. It is this structural diversity that grants flavones a wide range of biological activities [34]. Their extensive biological activity spectrum has attracted the interest of medicinal chemists, culminating in the discovery of several lead molecules for numerous pathologies. Alkylphenols and alkylmethoxyphenols hold significant commercial importance due to their alkyl groups, which range in size from one to twelve carbons [31]. The majority of alkylphenols are used to synthesize derivatives, with applications spanning from surfactants to pharmaceuticals. Hydroxycinnamic acid derivatives have diverse applications, particularly in cosmetics [35]. They exhibit photoprotective, antioxidant, and anti-inflammatory effects. Stilbenes, such as Piceatannal 3-O-glucoside, are known for their anti-cancer activity and their potential in preventing liver and lung diseases. There are currently two patents for the applications of this molecule in the prevention and treatment of pulmonary diseases and fatty liver syndrome [36]. Finally, tyrosolic derivatives possess antioxidant activity, making them suitable for use in supplements and cosmetic preparations [35]. P-HPEA-EDA is considered a promising compound for the prevention and therapy of colon cancer [37].

*P. nigdeneensis*, on the other hand, has a smaller variety of discriminating biochemical classes. We found alkylphenols, lignans, and phenolic terpenes. Alkylphenols have a significantly higher extraction yield in this species when using hexane as the extraction solvent. From a pharmaceutical perspective, lignans perform many biological functions, including antioxidant, antimicrobial, anticarcinogenic, antiplatelet aggregation, hormone modulation, and the detoxification of enzyme systems [38,39]. In particular, Arctigenin has several applications in the anti-cancer field for pancreas, colon, and stomach cancers [40,41], as well as anti-leukemic [42] and anti-inflammatory effects [43]. The last class is phenolic terpenes. These fall into the terpene category and exhibit varied effects, including cancer chemopreventive, antimicrobial, antifungal, antiviral, antihyperglycemic, anti-inflammatory, and anti-parasitic activities [44]. Terpenes are also known as stimulators of skin penetration and as agents involved in the prevention and treatment of various inflammatory diseases.

### 2.4. Antioxidant Properties

In the present study, while the n-hexane and dichloromethane extracts showed no DPPH scavenging activity, the ethyl acetate, ethanol, and water extracts did display DPPH scavenging activity (*P. brachiata:* 4.74–7.25 mg TE/g). Interestingly, for both *petrosimonia* species, the same order was obtained based on the DPPH scavenging ability (ethanol > water > ethyl acetate). On the other hand, all tested extracts of *P. brachiata* and *P. nigdeensis* exerted ABTS scavenging activity (30.56–84.22 mg TE/g and 16.12–98.02 mg TE/g, respectively). Remarkably, the same trend in ABTS scavenging ability was obtained for extracts of both species, whereby the more polar extracts showed a higher ABTS scavenging potential and the least polar/non-polar extracts showed a lower potential (water > ethanol > ethyl acetate > dichloromethane > n-hexane) (Table 5). The polarity-dependent increase in radical scavenging properties indicates the extraction of strong antioxidant compounds in polar solvents, as previously reported in other studies [45,46]. n-hexane and dichloromethane are non-polar solvents. Non-polar solvents extract lipids and either dissolve or destabilize the plant cell membrane, thereby facilitating the release of intracellular components [47]. They can extract particularly lipophilic compounds such as fatty acids or phytosterols. Since the lipophilic compounds have weaker antioxidant properties, the non-polar extracts have the weakest radical scavenging abilities for DPPH and ABTS [46,47]. All extracts acted as reducing agents in the CUPRAC (*P. brachiata*: 26.02–69.68 mg TE/g; *P. nigdeensis:* 36.01–80.35 mg TE/g) and FRAP (*P. brachiata*: 36.93–53.91 mg TE/g; *P. nigdeensis:* 31.70–67.69 mg TE/g) assays (Table 5). In the CUPRAC assay, the water extracts showed the least reducing activity, while the ethyl acetate extract of *P. brachiata* and the ethanol extract of *P. nigdeensis* showed the highest activity. On the other hand, the ethanol extracts of both species yielded the highest reducing activity in the FRAP assay. The phosphomolybdenum assay also revealed all extracts to possess total antioxidant capacity in the range of 0.16–2.17 mmol TE/g for *P. brachiata* and 0.24–1.95 mmol TE/g for *P. nigdeensis*. A metal chelating activity was also displayed by all extracts (*P. brachiata:* 14.74–30.52 mg EDTAE/g; *P. nigdeensis:* 18.43–33.80 mg EDTAE/g). The water extracts were found to possess the highest metal chelating power (Table 5). In general, the ethanol and water extracts of both *Petrosimonia* species showed a stronger radical scavenging and reducing ability than the other extracts. In particular, Table 2 shows that the ethanol and water extracts were rich in anthocyanins and flavonols, and these compounds can be attributed to the observed antioxidant abilities. Flavonols have been reported to be powerful antioxidant molecules, especially with the 3-OH group in the flavonoid ring, and this group is very effective in hydrogen and electron donation ability in antioxidant tests [48,49]. In addition, anthocyanins, with their three-ring structure, are able to improve the antioxidant properties of the tested extracts. The -OH groups function as hydrogen donors during redox reactions, while the -OCH_3_ groups are considered to provide an intramolecular electron donor effect [50].

The term antioxidant is one of the most popular in various scientific fields. Antioxidants are substances that are capable of hindering oxidation. They are known as free radical scavengers, which can either prevent the formation of reactive oxygen species or eliminate them before they can cause damage to vital cell components [51]. Therefore, an antioxidant diminishes the development and occurrence of different oxidative-induced pathological disorders such as diabetes, inflammation, aging, cancer, cataract, nephrotoxicity, neurodegenerative disorders, and liver and cardiovascular diseases [52]. Various analytical methods have been established to assess the antioxidant properties of plant-based phytochemicals. Indeed, their antioxidant activity rests on their chemical structure, along with their ability to donate hydrogen electrons, metal chelation, and their ability to delocalize the unpaired electron within the aromatic structure [53]. In this sense, the antioxidant properties of a plant extract are an important indicator for the development of functional applications, including pharmaceuticals, nutraceuticals, and cosmetics. To the best of our knowledge, there is very little information about the antioxidant properties of members of the genus *Petrosimonia*. In contrast to the results we presented, Asan-Ozusaglam et al. [25] reported that n-hexane had the highest DPPH radical scavenging ability. In addition, the authors found that the n-hexane extract exhibited the best metal chelating ability compared to methanol, ethanol, dichloromethane, and water. In the literature, some species, such as *Suaeda edulis* [54] and *Chenopodium ambrosioides* [55] from the family Chenopodiaceae, indicate significant antioxidant properties in in vitro test systems. From this point, our results can be valuable for the determination of new sources of antioxidants, and the tested *Petrosimonia* species can be considered as potential sources of natural antioxidants in the pharmaceutical, nutraceutical, and cosmeceutical fields. This study can therefore be an important starting point for further studies on the genus *Petrosimonia*.

### 2.5. Enzyme Inhibition Properties

Drugs functioning as enzyme inhibitors constitute an important segment of the orally bioavailable therapeutic agents that are currently in clinical use for treating diverse illnesses. Similarly, many of the drug discovery and development endeavors are focused on the identification and optimization of drug candidates that can act by inhibiting specific enzyme targets linked to different types of diseases [56,57,58].

Given that the cholinergic system plays a significant role in regulating learning and memory processes, it is considered as a pertinent target for the design of anti-Alzheimer’s drugs. Cholinesterase inhibitors improve cholinergic transmission by directly inhibiting the enzyme acetylcholinesterase (AChE) that hydrolyses acetylcholine. It has also been found that both acetylcholinesterase and butyrylcholinesterase (BChE) play a vital part in Aβ-aggregation during the initial phases of senile plaque formation. Consequently, AChE and BChE inhibition have been documented to be critical targets for the effective management of Alzheimer’s disease by a rise in the availability of acetylcholine in the brain areas and a decline in the Aβ deposition [59]. Additionally, cholinesterase inhibitors are the only available and approved treatment for Alzheimer’s disease and have been reported to stabilize or delay cognitive deterioration [60].

In the current study, extracts of both *P. brachiata* and *P. nigdeensis* species were found to inhibit acetylcholinesterase (0.24–3.77 mg GALAE/g and 0.16–3.85 mg GALAE/g, respectively). Moreover, with the exception of the water extracts, all other extracts of both species showed anti-butyrylcholinesterase activity (0.73–2.86 mg GALAE/g and 1.47–2.23 BChE mg GALAE/g). While the ethanolic and dichloromethane extracts of *P. brachiata* displayed the highest anti-AChE potential, the n-hexane and ethanol extracts of *P. nigdeensis* were found to have the most potent anti-AChE potential (Table 6).

Although several synthetic oral antidiabetic drugs are currently available, there is a critical need for the discovery and design of new antidiabetic drugs, given the development of resistance and adverse effects of those drugs for long-term usage. On the contrary, plants or herbal sources are becoming increasingly popular to the pharmaceutical industries worldwide, in a search for potential bioactive compounds for developing targeted novel antidiabetic drugs that can control diabetes with fewer undesirable effects than conventional drugs [61]. 

The inhibition of α-glucosidase and α-amylase, enzymes involved in carbohydrate digestion, can significantly lessen the postprandial increase in blood glucose and can, thus, be an imperative approach in the management of blood glucose levels in type 2 diabetic patients. In this regard, there has been a renewed interest in plant-based medicines modulating physiological effects in the treatment and prevention of diabetes and obesity [62].

In the present study, all extracts showed amylase inhibition (*P. brachiata*: 0.11–1.28 mmol ACAE/g; *P. nigdeensis*: 0.11–1.25 mmol ACAE/g). Moreover, all extracts except the water extracts demonstrated glucosidase inhibition (*P. brachiata*: 2.38–2.73 mmol ACAE/g; *P. nigdeensis*: 2.37–2.72 mmol ACAE/g) (Table 6).

Melanin is a major pigment of human skin that protects the skin from harmful ultraviolet radiation, DNA damage, and oxidative stress. However, an excessive buildup of melanin may lead to several hyperpigmentation-related diseases. Tyrosinase is a copper-containing enzyme that controls the rate-limiting step of melanin synthesis. Therefore, inhibiting tyrosinase is a key target for researchers in the search for a treatment against hyperpigmentation [63].

In plants, secondary metabolites such as flavonoids, stilbenes, chalcones, tannins, hydroquinone, and kojic acid, among others, have been revealed to exert anti-tyrosinase activity. Tyrosinase inhibitors of herbal origin have been widely reported to possess a potent inhibitory action on tyrosinase [64,65] and are greatly considered for research purposes. In this study, extracts of both species acted as tyrosinase inhibitors (*P. brachiata*: 43.30–60.58 mg KAE/g; *P. nigdeensis*: 36.74–61.40 mg KAE/g), except the water extracts. For both species, the ethanolic extracts followed by the ethyl acetate extracts showed the highest tyrosinase inhibitory effect (Table 6).

To the best of our knowledge, there is no information on the enzyme inhibitory properties of members of the genus *Petrosimonia*. Thus, the presented results are the first report of these effects and could provide a scientific basis for the genus. The results can also provide alternative raw materials for functional applications such as pharmaceuticals, nutraceuticals, and cosmeceuticals.

### 2.6. Pearson’s Correlation

Pearson’s coefficients were calculated in order to highlight the possible correlations between bioactive compounds and biological activities reported for the different Petrosimonia species. Phenolic compounds derived from *P. brachiata* leaf extracts (Supplementary Table S2) reported a high correlation with antioxidant and enzyme inhibition activities. Interestingly, low-molecular-weight compounds have been shown to possess a high positive correlation with CUPRAC, FRAP, and BChE activities (r > 0.7; *p* < 0.01), and a negative correlation with the metal chelating activity (r < −0.9; *p* < 0.01). While stilbenes and lignans reported a positive correlation with amylase (r > 0.7; *p* < 0.01), phenolic acids had a negative correlation with a-glucosidase (r = −0.708).

The relationship between antioxidant activity, evaluated through CUPRAP and FRAP assays, and low molecular weight compounds is well described in the literature [66,67]. The scavenging activity of LMW compounds depends on their structural features. The most represented class in the LMW group are tyrosyls, phenolic terpenes, and alkylphenols. The antioxidant effect of these classes of compounds is well described [68,69,70]. Lignans and stilbenes are correlated to a different modulation of enzyme inhibitor effects, particularly the inhibition of the α-amylase enzyme [71]. Regarding inhibition activities related to these classes of compounds, the accepted mechanism of action is related to the capability to maintain enzymes in their reduced states.

Considering *P. nigdeensis* (Supplementary Table S3), the pool of phenolics and flavonoids was reported to be positively correlated with antioxidant activities and inhibition activity. Anthocyanins were positively correlated with DPPH and FRAP activity (r > 0.8; *p* < 0.001). Flavonols had a positive correlation with FRAP (r > 0.7; *p* < 0.05). Lignans had a positive correlation with DPPH (r > 0.7 and *p* < 0.02), but they had a negative correlation with FRAP (r > −0.7; *p* < 0.02). Flavones and stilbenes had a positive correlation with the molybdenum assay (r > 0.7 and *p* < 0.001).

The positive correlation between different polyphenol classes and increased antioxidant activity is also important for *P. nigdeensis*. However, the results obtained show that different classes of compounds have more significant antioxidant effects. Among them, we find anthocyanins, flavonols, and lignans. The anthocyanins’ and flavonols’ activity related to the antioxidant activity has been an object of study for a long time [72,73,74]. The lignans’ antioxidant activity under different conditions, in particular, shows that lignans have the ability to generate stable products by giving electrons to free radicals and ROS [75]

## 3. Materials and Methods

### 3.1. Plant Materials

Plant materials were gathered from a field investigation in 2021 (Konya, Turkey) and the information on their locations is given below. Taxonomic identification, performed by Dr. Evren Yildiztugay, resulted in the deposition of a specimen in the herbarium of Selcuk University. The aerial parts were meticulously separated, dried in the shade at room temperature, ground into powder using a laboratory mill, and were stored in darkness.

*Petrosimonia nigdeensis* Aellen: Bolluk Lake (Cihanbeyli, Konya), Salty steppes, 950 m.

*Petrosimonia brachiata* (Pall.) Bunge: Koyuncu Salt Industry (Şereflikoçhisar, Ankara), Salty steppes, 920 m.

### 3.2. Extraction Method

A total of 10 g of dried plant material was accurately weighed and placed into a suitable container. In total, 200 mL of each solvent (n-hexane, ethyl acetate, dichloromethane, and ethanol, separately) was measured and added to separate containers holding the plant material. The extracts were stirred and underwent extraction overnight at room temperature. For the aqueous extract, the same amount of plant material (10 g) was used, and 200 mL of hot water was poured over the plant material, which was allowed to steep for 15 min. This process helps to extract water-soluble compounds effectively. After the extraction process, the organic solvents (n-hexane, ethyl acetate, dichloromethane, and ethanol) containing the plant extracts were subjected to evaporation to remove the solvents. To remove water, a freeze-drying technique was employed. The organic extracts were dissolved in ethanol, while the aqueous extracts were prepared in water before analysis.

### 3.3. Assay for Total Phenolic and Flavonoid Contents

The quantification of phenols and flavonoids was conducted in accordance with the procedures outlined in [76]. The Folin–Ciocalteu method was used for the quantification of the total phenolic content, and the results were expressed as gallic acid equivalents (mg GAE/g). Regarding the total flavonoid content, AlCl_3_ was used to evaluate the total flavonoid content in the extracts, and the results were given as mg rutin equivalent RE/g. The experimental details are given in the Supplementary Materials.

### 3.4. Untargeted Phenolic Compound Profiling using UHPLC QTOF-MS Spectrometry

Lyophilized samples were solubilized in 5 different solvent solutions (water, ethanol, ethyl acetate, dichloromethane, and n-hexane) using a dilution factor of 1:10. After the solubilization, samples were centrifugated for 10 min at 6000× *g* in a refrigerated centrifuge (Eppendorf 5430R, Hamburg, Germany), and the supernatants were collected and filtrated trough 0.22 µm cellulose filters in amber vials. For the untargeted phenolic profiling, samples were analyzed with ultra-high-pressure liquid chromatography coupled to a quadrupole time-of-flight mass spectrometer (UHPLC-ESI/QTOF-MS; Agilent Technologies, Santa Clara, CA, USA). The analytical conditions have been well described in our previous work [77]. Chromatographic separation was achieved in reverse phase using a C18 column (Agilent Zorback Eclipse Plus 4.6 mm × 150 mm, nominal surface area of 160 m^2^/g and a controlled pore size of 95 Å) through a water–acetonitrile gradient elution (6–94% in 33 min). Positive full scan mode was used to acquire an accurate mass in the 100–1200 *m*/*z* range at a rate of 0.8 spectra/s. The injection volume was 6 μL, and three replicates were considered for each extract condition. After data acquisition, compound identifications were conducted using the software Profinder B.08 (from Agilent Technologies), according to the ‘find-by-formula’ algorithm against the Phenol-explorer 3.6 database [78]. The annotation workflow allowed compound identification according to Level 2 confidence (i.e., putatively annotated compounds), with reference to the COSMOS Metabolomics Standards Initiative. Afterward, cumulative intensities per class of compounds were calculated and qualitative concentrations were determined using calibration curves of pure individual standard compounds (expressed as mg equivalents/g dry weight (dw); Extrasynthese, Lyon, France; purity > 98%) analyzed under the same conditions: ferulic acid (phenolic acids), quercetin (flavonols), sesamin (lignans), cyanidin (anthocyanins), catechin (flavan-3-ols), luteolin (flavones and other remaining flavonoids), resveratrol (stilbenes), and tyrosol (tyrosols and other polyphenols).

### 3.5. Biological Activity Assays

Antioxidant tests were conducted in vitro following known protocols outlined in previous research [79]. The results of the DPPH, ABTS, CUPRAC, and FRAP assays were expressed in milligrams of Trolox equivalents (TE) per gram of extract. The antioxidant capacity was measured using the phosphomolybdenum (PBD) assay and the results were expressed as mmol of Trolox equivalents (TE) per gram of extract. The metal chelating activity (MCA) was quantified in milligrams of disodium edetate equivalents (EDTAEs) per gram of extract. [79]. Enzyme inhibition investigations were carried out on the materials using established methods. The enzyme inhibition for amylase and glucosidase was quantified in mmol of acarbose equivalents (ACAEs) per gram of extract, while acetylcholinesterase (AChE) and butyrylcholinesterase (BChE) activity inhibition was expressed in mg of galanthamine equivalents (GALAEs) per gram of extract. The tyrosinase inhibition was measured in milligrams of kojic acid equivalents (KAEs) per gram of the tested extracts. The experimental details are given in the Supplementary Materials.

### 3.6. Statistical Analysis

A one-way analysis of variance (ANOVA with Tukey’s post hoc test) was performed considering data from each assay and using SPSS 26.0 software (SPSS Inc., Chicago, IL, USA). The chemometrics analysis was conducted using Mass Profiler Professional B12.6 (Agilent Technologies, Santa Clara, CA, USA), and data normalization was performed according to previously published work [80]. Specifically, identified compounds were filtered by frequency, normalized at the 75th percentile, and were baselined with the median of all samples. The resulting dataset was investigated through both unsupervised hierarchical cluster analysis (HCA) and supervised Orthogonal Projections to Latent Structures Discriminant Analysis (OPLS-DA; SIMCA 16, Umetrics, Malmo, Sweden) approaches. The OPLS-DA models of leaves and roots were successively cross-validated (CV-ANOVA; *p* < 0.01) and permutation testing was conducted after inspecting the model parameters (goodness-of-fit, R^2^Y, and goodness-of-prediction, Q^2^Y) and model outliers (Hotelling’s T2). The variable importance in projection (VIP) metabolites, having a score >1.2, were extrapolated as being the biomarker responsible for the discrimination capacity of the models.

## 4. Conclusions

The metabolomic and biochemical results of this study highlighted the differences between the polyphenolic profiles of the two species. The extracts obtained had different antioxidant and enzyme inhibition properties, and the solvent type used was shown to influence the pharmacological properties of the extracts. This study, through metabolomic and biochemical assays, has helped to gather information on the in vitro pharmacological properties and phytochemical contents of two species of *Petrosimonia*. While the different extracts showed varied TPC and TFC, the polar extracts showed better radical scavenging abilities in the DPPH and ABTS assays, the ethanol and/or ethyl acetate extracts demonstrated a better reducing activity, and the water extracts displayed a higher metal chelating power. On the other hand, the water extracts were revealed to be poor inhibitors of the tested enzymes, while mostly the ethanol extracts showed a more potent enzyme inhibition. Overall, the findings obtained in this study demonstrated the antioxidant and enzyme inhibitory effect of these two halophytic species and that the different solvents had an effect on the biological potency of the plant species. From this point, the tested *Petrosimonia* species can be considered as potential sources of bioactive compounds in the preparation of health-promoting applications, for example, in Alzheimer’s disease or diabetes. However, we strongly recommend further studies to understand their cytotoxic effects and pharmacokinetic properties.

## Figures and Tables

**Table 1:** Total phenolic and flavonoid contents of the tested extracts.

Species	Extracts	Total Phenolic Content (mg GAE/g)	Total Flavonoid Content (mg RE/g)
*Petrosimonia* *brachiata*	n-hexane	16.66 ± 0.16	11.46 ± 0.34
	Dichloromethane	20.18 ± 0.81	4.56 ± 0.22
	Ethyl acetate	23.91 ± 0.36	3.86 ± 0.31
	Ethanol	20.65 ± 0.07	18.99 ± 0.08
	Water	11.14 ± 0.17	5.99 ± 0.04
*Petrosimonia nigdeensis*	n-hexane	19.89 ± 0.75	8.80 ± 0.17
	Dichloromethane	20.45 ± 0.89	7.08 ± 0.29
	Ethyl acetate	21.31 ± 0.69	12.54 ± 0.18
	Ethanol	24.22 ± 0.13	22.03 ± 0.35
	Water	14.09 ± 0.11	3.15 ± 0.02

Values are reported as mean ± SD of three parallel measurements. GAE: gallic acid equivalents; RE: rutin equivalents.

**Table 2:** Polyphenols’ semi quantifications.

Species	Extracts	Antocyanins (µg/g)	Flavones (µg/g)	Flavonols (µg/g)	Ph.Acids (µg/g)	LMW (µg/g)	Lignans (µg/g)	Stilbenes (µg/g)
*P. brachiata*	n-hexane	26.45 ± 18.44 b	4.49 ± 3.02 a	n/d	16.46 ± 1.86 d	13.40 ± 6.66 c	5.34 ± 1.63 bc	3.09 ± 0.27 c
	Dichloromethane	n/d	1.87 ± 0.71 c	n/d	51.07 ± 16.42 b	55.26 ± 2.33 b	10.11 ± 2.82 a	16.91 ± 0.36 a
	Ethyl acetate	n/d	1.60 ± 0.11 c	n/d	19.34 ± 0.78 cd	56.02 ± 5.99 ab	7.61 ± 0.91 ab	11.75 ± 1.36 b
	Ethanol	44.91 ± 1.04 a	2.79 ± 0.22 ac	8.00 ± 0.45 a	44.88 ± 10.66 bc	63.87 ± 1.60 a	1.5 ± 0.58 d	2.88 ± 0.07 c
	Water	31.8 ± 9.62 b	4.62 ± 1.71 ab	0.79 ± 0.15 b	308.67 ± 137.99 a	18.12 ± 2.72 c	2.92 ± 0.34 cd	2.99 ± 0.22 c
Significance	***	**	***	***	***	***	***	***
*P. nigdeensis*	n-hexane	n/d	0.81 ± 0.029 d	n/d	15.01 ± 6.28	99.05 ± 7.43 a	11.22 ± 4.04 a	0.69 ± 0.6 d
	Dichloromethane	7.54 ± 0.48 c	9.45 ± 0.139 a	1.36 ± 0.14 d	16.37 ± 9.8	54.74 ± 2.57 b	8.16 ± 2.03 ab	33.10 ± 0.32 a
	Ethyl acetate	3.32 ± 0.26 d	7.95 ± 0.10 b	2.39 ± 0.09 c	23.35 ± 6.66	100.42 ± 12.92 a	6.16 ± 1.52 b	26.03 ± 0.51 b
	Ethanol	61.02 ± 1.4 a	5.93 ± 0.156 c	28.08 ± 0.22 a	23.89 ± 5.79	55.56 ± 3.95 b	n/d	9.21 ± 0.42 c
	Water	32.7 ± 0.82 b	0.102 ± 0.1 e	6.18 ± 0.23 b	23.28 ± 3.17	24.57 ± 2.85 c	6.92 ± 0.04 b	1.05 ± 0.06 d
Significance		***	***	***	n.s.	***	**	***

Semi-quantitative analysis of different phenolic subclasses in *P. brachiata* and *P. nigdeensis*. Values are expressed as three replicates’ mean concentration (µg g^−1^ dry matter). The letters in the same phenolic subclass indicate significant differences between treatments (ANOVA and Tukey’s HSD post hoc test, *p*-value < 0.05). Abbreviations: LMW: low-molecular-weight phenolic compounds. *** *p* value < 0.001; ** *p* value < 0.01.

**Table 3:** VIP markers were identified for the discrimination of different solvent extraction methods on *P. brachiata* leaves. Discriminant phenolic compounds are provided with their compound classification, VIP scores ± standard errors (VIP ≥ 1.20), and log fold change values obtained from a pairwise comparison between different solvents and the control (water). The extraction solvents are dichloromethane (DCM), ethyl acetate (EA), ethanol (EtOH), and n-hexane.

Primary ID	Class	Sub-Class	VIP Score ± SE	Log FC [DCM] vs. [H2O]	Log FC [EA] vs. [H2O]	Log FC [EtOH] vs. [H2O]	Log FC [Hexane] vs. [H2O]
Gardenin B	Flavonoids	Flavones	1.22 ± 1.37				
4-Vinylguaiacol	Other polyphenols	Alkylmethoxyphenols	1.20 ± 0.45	6	6		
5-Pentadecylresorcinol		Alkylphenols	1.82 ± 0.67		6		
1,4-Naphtoquinone		Naphtoquinone	1.28 ± 0.17	−0.3	−0.89	−1.01	−6
p-HPEA-EDA		Tyrosols	1.60 ± 0.47		6		
Hydroxytyrosol			1.28 ± 0.17	−0.3	−0.89	−1.01	−6
Cinnamolyl Glucose	Phenolic acid	Hydroxycinnamic acids	1.22 ± 0.29	6	6		
Piceatannal 3-O-glucoside	Stilbenes	Stilbenes	1.29 ± 0.76	6	6		25.5

**Table 4:** VIP markers were identified for the discrimination of different solvent extraction methods on *P. nigdeensis* leaves. Discriminant phenolic compounds are provided with their compound classification, VIP scores ± standard errors (VIP ≥ 1.20), and log fold change values obtained from a pairwise comparison between different solvents and the control (water). The extraction solvents are dichloromethane (DCM), ethyl acetate (EA), ethanol (EtOH), and n-hexane.

Primary ID	Class	Sub-Class	VIP Score ± SE	Log FC [DCM] vs. [H2O]	Log FC [EA] vs. [H2O]	Log FC [EtO] vs. [H2O]	Log FC [Hexane] vs. [H2O]
Arctigenin	Lignans	Lignans	1.22 ± 0.44				28.5
5-Heneicosenylresorcinol	Other polyphenols	Alkylphenols	1.23 ± 0.41	6		6	28.5
5-Heneicosylresorcinol			1.21 ± 0.32		6	6	28.5
Rosmadial		Phenolic terpenes	1.27 ± 0.46				28.5

**Table 5:** Antioxidant properties of the tested extracts.

Species	Extracts	DPPH (mg TE/g)	ABTS (mg TE/g)	CUPRAC (mg TE/g)	FRAP (mg TE/g)	PBD (mmol TE/g)	MCA (mg EDTAE/g)
*P. brachiata*	n-hexane	na	30.56 ± 1.27	53.64 ± 2.70	41.66 ± 1.21	2.17 ± 0.10	28.81 ± 0.98
	Dichloromethane	na	37.42 ± 0.71	60.19 ± 2.02	42.68 ± 1.22	1.88 ± 0.03	22.72 ± 0.25
	Ethyl acetate	4.74 ± 0.40	59.94 ± 2.30	69.68 ± 0.91	46.59 ± 0.30	1.89 ± 0.03	21.45 ± 0.66
	Ethanol	7.25 ± 0.95	69.81 ± 1.53	64.69 ± 1.79	53.91 ± 0.32	1.28 ± 0.02	14.74 ± 0.86
	Water	6.14 ± 0.64	84.22 ± 0.93	26.02 ± 0.55	36.93 ± 0.45	0.16 ± 0.04	30.52 ± 0.21
*P. nigdeensis*	n-hexane	na	16.12 ± 2.12	49.51 ± 1.14	31.70 ± 0.97	1.26 ± 0.03	18.90 ± 3.20
	Dichloromethane	na	41.37 ± 1.37	63.05 ± 1.12	44.23 ± 0.27	1.79 ± 0.11	23.33 ± 0.87
	Ethyl acetate	6.93 ± 1.16	56.65 ± 4.30	65.58 ± 0.96	47.59 ± 0.99	1.95 ± 0.17	25.01 ± 0.48
	Ethanol	16.55 ± 1.14	78.64 ± 1.95	80.35 ± 3.28	67.69 ± 0.63	1.50 ± 0.08	18.43 ± 0.42
	Water	15.29 ± 0.25	98.02 ± 0.94	36.01 ± 0.23	49.63 ± 0.39	0.24 ± 0.01	33.80 ± 0.03

Values are reported as mean ± SD of three parallel measurements. PBD: phosphomolybdenum; MCA: metal chelating activity; TE: Trolox equivalent; EDTAE: EDTA equivalent; na: not active.

**Table 6:** Enzyme inhibitory effects of the tested extracts.

Species	Extracts	AChE (mg GALAE/g)	BChE (mg GALAE/g)	Tyrosinase (mg KAE/g)	Amylase (mmol ACAE/g)	Glucosidase (mmol ACAE/g)
*P. brachiata*	n-hexane	2.77 ± 0.11	0.73 ± 0.02	47.43 ± 2.21	1.01 ± 0.02	2.63 ± 0.10
	Dichloromethane	3.15 ± 0.14	1.44 ± 0.09	43.30 ± 3.40	1.26 ± 0.02	2.38 ± 0.09
	Ethyl acetate	2.84 ± 0.04	1.56 ± 0.09	48.07 ± 0.88	1.28 ± 0.01	2.50 ± 0.05
	Ethanol	3.77 ± 0.03	2.86 ± 0.10	60.58 ± 1.07	0.72 ± 0.03	2.73 ± 0.02
	Water	0.24 ± 0.04	na	na	0.11 ± 0.01	na
*P. nigdeensis*	n-hexane	3.85 ± 0.19	1.73 ± 0.07	36.74 ± 0.55	0.90 ± 0.01	2.37 ± 0.16
	Dichloromethane	3.27 ± 0.31	1.47 ± 0.06	49.65 ± 1.09	1.25 ± 0.01	2.42 ± 0.05
	Ethyl acetate	2.98 ± 0.15	2.23 ± 0.02	57.69 ± 0.80	1.22 ± 0.01	2.42 ± 0.07
	Ethanol	3.76 ± 0.07	2.19 ± 0.05	61.40 ± 2.39	0.89 ± 0.01	2.72 ± 0.0
	Water	0.16 ± 0.01	na	na	0.11 ± 0.01	na

Values are reported as mean ± SD of three parallel measurements. GALAE: galantamine equivalent; KAE: kojic acid equivalent; ACAE: acarbose equivalent; na: not active.

## Data Availability

Data are contained within the article and Supplementary Materials.

## Supplementary Materials

The following supporting information can be downloaded at: https://www.mdpi.com/article/10.3390/plants13152073/s1, Table S1: Metabolomics raw data; Table S2: Pearson’s correlation for *P. branchiata*; Table S3: Pearson’s correlation for *P. nigdeensis*. Reference [81] is cited in the Supplementary Materials.

## References

[1] Grozeva N., Todorova M., Pavlov D. (2019). Karyological and morphological variation within *Petrosimonia brachiata* Bunge in Bulgaria. Bot. Serbica.

[2] Ferreira M.J., Pinto D.C., Cunha Â., Silva H. (2022). Halophytes as medicinal plants against human infectious diseases. Appl. Sci..

[3] Buhmann A., Papenbrock J. (2013). An economic point of view of secondary compounds in halophytes. Funct. Plant Biol..

[4] Khan M., Musharaf S., Shinwari Z.K. (2011). Ethnobotanical importance of halophytes of Noshpho salt mine, District Karak, Pakistan. Res. Pharm. Biotechnol..

[5] Ksouri R., Ksouri W.M., Jallali I., Debez A., Magné C., Hiroko I., Abdelly C. (2012). Medicinal halophytes: Potent source of health promoting biomolecules with medical, nutraceutical and food applications. Crit. Rev. Biotechnol..

[6] Qasim M., Gulzar S., Khan M.A. (2011). Halophytes as medicinal plants. Urban. Land Use Land Degrad. Environ..

[7] Rodrigues M.J., Pereira C.G., Oliveira M., Zengin G., Custódio L. (2023). Salt-Tolerant Plants as Sources of Antiparasitic Agents for Human Use: A Comprehensive Review. Marine Drugs.

[8] Grigore M.-N., Oprica L. (2015). Halophytes as possible source of antioxidant compounds, in a scenario based on threatened agriculture and food crisis. Iran. J. Public Health.

[9] Davis P. (2019). Flora of Turkey, Volume 2.

[10] Nurpeisova D., Toktarbek M., Seitimova G., Yeskaliyeva B., Burasheva G.S., Choudhary M.I. (2018). Phytochemical analysis of *Petrosimonia sibirica* grown in Kazakhstan. Int. J. Biol. Chem..

[11] Sun W., Ma Z., Zhang X., Yang H., Sun W. (2015). Secondary metabolites of *Petrosimonia sibirica*. Chem. Nat. Compd..

[12] Toktarbek M., Seitimova G., Eskalieva B., Burasheva G.S., Beyatli A., Choudhary M.I. (2019). Sterols and Flavonoids from the Pelitohalophytes *Petrosimonia glaucescens* and *Climacoptera brachiata*. Chem. Nat. Compd..

[13] Toktarbek M., Seitimova G., Yeskaliyeva B., Burasheva G.S., Choudhary M.I. (2021). Phenolic Compounds from the Plant *Petrosimonia triandra*. Chem. Nat. Compd..

[14] Podar D., Macalik K., Réti K.-O., Martonos I., Török E., Carpa R., Weindorf D.C., Csiszár J., Székely G. (2019). Morphological, physiological and biochemical aspects of salt tolerance of halophyte *Petrosimonia triandra* grown in natural habitat. Physiol. Mol. Biol. Plants.

[15] Székely G., Szígyártó N.Z., Tóth A., Barta C. (2022). The Rhizosphere of *Petrosimonia triandra* may Possess Growth-Inducing and Salinity-Tolerance Potential. Hung. J. Ind. Chem..

[16] Todorova M., Grozeva N., Pleskuza L., Yaneva Z., Gerdgikova M. (2014). Relationship between soil salinity and *Bassia hirsuta*, *Salicornia europaea* agg. and *Petrosimonia brachyata* distribution on the territory of Pomorie lake and Atanasovsko lake. Agric. Sci. Technol..

[17] Shehab N.G., Abu-Gharbieh E., Bayoumi F.A. (2015). Impact of phenolic composition on hepatoprotective and antioxidant effects of four desert medicinal plants. BMC Complement Altern. Med..

[18] Petrosimonia brachiata—Uses, Benefits & Care. https://www.selinawamucii.com/plants/amaranthaceae/petrosimonia-brachiata/.

[19] John B., Sulaiman C., George S., Reddy V. (2014). Total phenolics and flavonoids in selected medicinal plants from Kerala. Int. J. Pharm. Pharm. Sci.

[20] Nabavi S., Ebrahimzadeh M., Nabavi S., Hamidinia A., Bekhradnia A. (2008). Determination of antioxidant activity, phenol and flavonoids content of *Parrotia persica* Mey. Pharmacologyonline.

[21] Ribarova F., Atanassova M., Marinova D., Ribarova F., Atanassova M. (2005). Total phenolics and flavonoids in Bulgarian fruits and vegetables. JU Chem. Metal.

[22] Thouri A., Chahdoura H., El Arem A., Omri Hichri A., Ben Hassin R., Achour L. (2017). Effect of solvents extraction on phytochemical components and biological activities of Tunisian date seeds (var. Korkobbi and Arechti). BMC Complement. Altern. Med..

[23] Addai Z.R., Abdullah A., Mutalib S.A. (2013). Effect of extraction solvents on the phenolic content and antioxidant properties of two papaya cultivars. J. Med. Plants Res..

[24] Dirar A., Alsaadi D., Wada M., Mohamed M., Watanabe T., Devkota H. (2019). Effects of extraction solvents on total phenolic and flavonoid contents and biological activities of extracts from Sudanese medicinal plants. S. Afr. J. Bot..

[25] Asan-Ozusaglam M., Erzengin M., Darilmaz D.O., Erkul S.K., Teksen M., Karakoca K. (2015). Antimicrobial and antioxidant activity of various solvent extracts of *Salsola stenoptera* Wagenitz and *Petrosimonia nigdeensis* Aellen (Chenopodiaceae) plants. Chiang Mai J. Sci..

[26] Belwal T., Ezzat S.M., Rastrelli L., Bhatt I.D., Daglia M., Baldi A., Devkota H.P., Orhan I.E., Patra J.K., Das G. (2018). A critical analysis of extraction techniques used for botanicals: Trends, priorities, industrial uses and optimization strategies. TrAC Trends Anal. Chem..

[27] Sánchez-Rangel J.C., Benavides J., Heredia J.B., Cisneros-Zevallos L., Jacobo-Velázquez D.A. (2013). The Folin–Ciocalteu assay revisited: Improvement of its specificity for total phenolic content determination. Anal. Methods.

[28] Brglez Mojzer E., Knez Hrnčič M., Škerget M., Knez Ž., Bren U. (2016). Polyphenols: Extraction Methods, Antioxidative Action, Bioavailability and Anticarcinogenic Effects. Molecules.

[29] Manach C., Williamson G., Morand C., Scalbert A., Rémésy C. (2005). Bioavailability and bioefficacy of polyphenols in humans. I. Review of 97 bioavailability studies. Am. J. Clin. Nutr..

[30] Hendrich A.B. (2006). Flavonoid-membrane interactions: Possible consequences for biological effects of some polyphenolic compounds1. Acta Pharmacol. Sin..

[31] Lorenc J.F., Lambeth G., Scheffer W. (1947). Alkylphenols. Kirk-Othmer Encyclopedia of Chemical Technology.

[32] Singh M., Kaur M., Silakari O. (2014). Flavones: An important scaffold for medicinal chemistry. Eur. J. Med. Chem..

[33] Cao G., Sofic E., Prior R.L. (1997). Antioxidant and prooxidant behavior of flavonoids: Structure-activity relationships. Free Radic. Biol. Med..

[34] Chen Z.Y., Chan P.T., Ho K.Y., Fung K.P., Wang J. (1996). Antioxidant activity of natural flavonoids is governed by number and location of their aromatic hydroxyl groups. Chem. Phys. Lipids.

[35] Zhou D.-Y., Sun Y.-X., Shahidi F. (2017). Preparation and antioxidant activity of tyrosol and hydroxytyrosol esters. J. Funct. Foods.

[36] The National Center for Biotechnology Information PubChem Patent Summary for US-2021196736-A1. https://pubchem.ncbi.nlm.nih.gov/patent/US-2021196736-A1.

[37] Khanal P., Oh W.K., Yun H.J., Namgoong G.M., Ahn S.G., Kwon S.M., Choi H.K., Choi H.S. (2011). p-HPEA-EDA, a phenolic compound of virgin olive oil, activates AMP-activated protein kinase to inhibit carcinogenesis. Carcinogenesis.

[38] Kiyama R. (2016). Biological effects induced by estrogenic activity of lignans. Trends Food Sci. Technol..

[39] Ram J., Moteriya P., Chanda S. (2015). Phytochemical screening and reported biological activities of some medicinal plants of Gujarat region. J. Pharmacogn. Phytochem..

[40] Lee D.-Y., Song M.-C., Yoo K.-H., Bang M.-H., Chung I.-S., Kim S.-H., Kim D.-K., Kwon B.-M., Jeong T.-S., Park M.-H. (2007). Lignans from the fruits of *Cornus kousa* Burg. and their cytotoxic effects on human cancer cell lines. Arch. Pharmacal Res..

[41] Takasaki M., Konoshima T., Komatsu K., Tokuda H., Nishino H. (2000). Anti-tumor-promoting activity of lignans from the aerial part of *Saussurea medusa*. Cancer Lett..

[42] Hirano T., Gotoh M., Oka K. (1994). Natural flavonoids and lignans are potent cytostatic agents against human leukemic HL-60 cells. Life Sci..

[43] Cho J.Y., Kim A.R., Yoo E.S., Baik K.U., Park M.H. (1999). Immunomodulatory Effect of Arctigenin, a Lignan Compound, on Tumour Necrosis Factor-α and Nitric Oxide Production, and Lymphocyte Proliferation. J. Pharm. Pharmacol..

[44] Paduch R., Kandefer-Szerszeń M., Trytek M., Fiedurek J. (2007). Terpenes: Substances useful in human healthcare. Arch. Immunol. Et Ther. Exp..

[45] Barchan A., Bakkali M., Arakrak A., Pagán R., Laglaoui A. (2014). The effects of solvents polarity on the phenolic contents and antioxidant activity of three Mentha species extracts. Int. J. Curr. Microbiol. Appl. Sci..

[46] Nawaz H., Shad M.A., Rehman N., Andaleeb H., Ullah N. (2020). Effect of solvent polarity on extraction yield and antioxidant properties of phytochemicals from bean (*Phaseolus vulgaris*) seeds. Braz. J. Pharm. Sci..

[47] Agatonovic-Kustrin S., Gegechkori V., Kustrin E., Morton D.W. (2024). The effect of lactic acid fermentation on the phytochemical content of fig leaf extracts compared to single solvent and sequential solvents extraction. S. Afr. J. Bot..

[48] Procházková D., Boušová I., Wilhelmová N. (2011). Antioxidant and prooxidant properties of flavonoids. Fitoterapia.

[49] Heim K.E., Tagliaferro A.R., Bobilya D.J. (2002). Flavonoid antioxidants: Chemistry, metabolism and structure-activity relationships. J. Nutr. Biochem..

[50] Garcia C., Blesso C.N. (2021). Antioxidant properties of anthocyanins and their mechanism of action in atherosclerosis. Free. Radic. Biol. Med..

[51] Hatwalne M.S. (2012). Free radical scavengers in anaesthesiology and critical care. Indian J. Anaesth..

[52] Neha K., Haider M.R., Pathak A., Yar M.S. (2019). Medicinal prospects of antioxidants: A review. Eur. J. Med. Chem..

[53] Kotha R.R., Tareq F.S., Yildiz E., Luthria D.L. (2022). Oxidative stress and antioxidants—A critical review on in vitro antioxidant assays. Antioxidants.

[54] Costa-Becheleni F.R., Troyo-Diéguez E., Ruiz-Hernández A.A., Ayala-Niño F., Bustamante-Salazar L.A., Medel-Narváez A., Martínez-Rincón R.O., Robles-Sánchez R.M. (2024). Determination of bioactive compounds and antioxidant capacity of the halophytes *Suaeda edulis* and *Suaeda esteroa* (Chenopodiaceae): An option as novel healthy agro-foods. Aims Agric. Food.

[55] Drioua S., El-Guourrami O., Assouguem A., Ameggouz M., Kara M., Ullah R., Bari A., Zahidi A., Skender A., Benzeid H. (2024). Phytochemical study, antioxidant activity, and dermoprotective activity of *Chenopodium ambrosioides* (L.). Open Chem..

[56] Copeland R.A., Harpel M.R., Tummino P.J. (2007). Targeting enzyme inhibitors in drug discovery. Expert Opin. Ther. Targets.

[57] Geronikaki A., Eleutheriou P.T. (2023). Enzymes and Enzyme Inhibitors—Applications in Medicine and Diagnosis. Int. J. Mol. Sci..

[58] Ouertani A., Neifar M., Ouertani R., Masmoudi A.S., Mosbah A., Cherif A. (2019). Effectiveness of enzyme inhibitors in biomedicine and pharmacotherapy. Adv. Tissue Eng. Regen. Med. Open Access.

[59] Anand P., Singh B. (2013). A review on cholinesterase inhibitors for Alzheimer’s disease. Arch. Pharmacal Res..

[60] Moreta M.P.-G., Burgos-Alonso N., Torrecilla M., Marco-Contelles J., Bruzos-Cidón C. (2021). Efficacy of acetylcholinesterase inhibitors on cognitive function in Alzheimer’s disease. Review of reviews. Biomedicines.

[61] Alam S., Sarker M.M.R., Sultana T.N., Chowdhury M.N.R., Rashid M.A., Chaity N.I., Zhao C., Xiao J., Hafez E.E., Khan S.A. (2022). Antidiabetic phytochemicals from medicinal plants: Prospective candidates for new drug discovery and development. Front. Endocrinol..

[62] Tundis R., Loizzo M.R., Menichini F. (2010). Natural products as α-amylase and α-glucosidase inhibitors and their hypoglycaemic potential in the treatment of diabetes: An update. Mini Rev. Med. Chem..

[63] Srivastava J., Mal J., Verma M., Sinha R. Mini-review on Inhibitors of Human Tyrosinase. Proceedings of the Conference BioSangam 2022: Emerging Trends in Biotechnology (BIOSANGAM 2022).

[64] Mukherjee P.K., Biswas R., Sharma A., Banerjee S., Biswas S., Katiyar C. (2018). Validation of medicinal herbs for anti-tyrosinase potential. J. Herb. Med..

[65] Riaz R., Batool S., Zucca P., Rescigno A., Peddio S., Saleem R.S. (2021). Plants as a promising reservoir of tyrosinase inhibitors. Mini-Rev. Org. Chem..

[66] Stoia M., Oancea S. (2022). Low-Molecular-Weight Synthetic Antioxidants: Classification, Pharmacological Profile, Effectiveness and Trends. Antioxidants.

[67] Geun Lee H., Jayawardena T.U., Liyanage N.M., Song K.M., Choi Y.S., Jeon Y.J., Kang M.C. (2022). Antioxidant potential of low molecular weight fucoidans from Sargassum autumnale against H_2_O_2_-induced oxidative stress in vitro and in zebrafish models based on molecular weight changes. Food Chem..

[68] Nagarajan S., Nagarajan R., Kumar J., Salemme A., Togna A.R., Saso L., Bruno F. (2020). Antioxidant Activity of Synthetic Polymers of Phenolic Compounds. Polymers.

[69] Casadey R., Challier C., Altamirano M., Spesia M.B., Criado S. (2021). Antioxidant and antimicrobial properties of tyrosol and derivative-compounds in the presence of vitamin B2. Assays of synergistic antioxidant effect with commercial food additives. Food Chem..

[70] Karković Marković A., Torić J., Barbarić M., Jakobušić Brala C. (2019). Hydroxytyrosol, Tyrosol and Derivatives and Their Potential Effects on Human Health. Molecules.

[71] Akshatha J., SantoshKumar H., Prakash H., Nalini M. (2021). In silico docking studies of α-amylase inhibitors from the anti-diabetic plant Leucas ciliata Benth. and an endophyte, *Streptomyces longisporoflavus*. 3 Biotech.

[72] Peng Y., Zhang H., Liu R., Mine Y., McCallum J., Kirby C., Tsao R. (2016). Antioxidant and anti-inflammatory activities of pyranoanthocyanins and other polyphenols from staghorn sumac (*Rhus hirta* L.) in Caco-2 cell models. J. Funct. Foods.

[73] Banach M., Wiloch M., Zawada K., Cyplik W., Kujawski W. (2020). Evaluation of Antioxidant and Anti-Inflammatory Activity of Anthocyanin-Rich Water-Soluble Aronia Dry Extracts. Molecules.

[74] Speisky H., Shahidi F., Costa de Camargo A., Fuentes J. (2022). Revisiting the Oxidation of Flavonoids: Loss, Conservation or Enhancement of Their Antioxidant Properties. Antioxidants.

[75] Polat Kose L., Gulcin İ. (2021). Evaluation of the Antioxidant and Antiradical Properties of Some Phyto and Mammalian Lignans. Molecules.

[76] Slinkard K., Singleton V.L. (1977). Total phenol analysis: Automation and comparison with manual methods. Am. J. Enol. Vitic..

[77] Arriola N.D.A., Chater P.I., Wilcox M., Lucini L., Rocchetti G., Dalmina M., Pearson J.P., Amboni R.D.d.M.C. (2019). Encapsulation of stevia rebaudiana Bertoni aqueous crude extracts by ionic gelation–Effects of alginate blends and gelling solutions on the polyphenolic profile. Food Chem..

[78] Rothwell J.A., Perez-Jimenez J., Neveu V., Medina-Remón A., M’Hiri N., García-Lobato P., Manach C., Knox C., Eisner R., Wishart D.S. (2013). Phenol-Explorer 3.0: A major update of the Phenol-Explorer database to incorporate data on the effects of food processing on polyphenol content. Database.

[79] Grochowski D.M., Uysal S., Aktumsek A., Granica S., Zengin G., Ceylan R., Locatelli M., Tomczyk M. (2017). In vitro enzyme inhibitory properties, antioxidant activities, and phytochemical profile of *Potentilla thuringiaca*. Phytochem. Lett..

[80] Zhang L., Rocchetti G., Zengin G., Ak G., Saber F.R., Montesano D., Lucini L. (2021). The UHPLC-QTOF-MS phenolic profiling and activity of Cydonia oblonga Mill. reveals a promising nutraceutical potential. Foods.

[81] Uysal S., Zengin G., Locatelli M., Bahadori M.B., Mocan A., Bellagamba G., De Luca E., Mollica A., Aktumsek A. (2017). Cytotoxic and enzyme inhibitory potential of two Potentilla species (*P. speciosa* L. and *P. reptans* Willd.) and their chemical composition. Front. Pharmacol..

